# Impact of Gender on the Association of Epicardial Fat Thickness, Obesity, and Circadian Blood Pressure Pattern in Hypertensive Patients

**DOI:** 10.1155/2015/924539

**Published:** 2015-04-30

**Authors:** In Kyoung Shim, Kyoung-Im Cho, Hyun-Su Kim, Jung-Ho Heo, Tae Joon Cha

**Affiliations:** Division of Cardiology, Department of Internal Medicine, Kosin University College of Medicine, Busan 602-702, Republic of Korea

## Abstract

This study aimed to investigate the effects of gender on the association between epicardial fat thickness (EFT) and circadian blood pressure (BP) changes in patients with recently diagnosed essential hypertension (EH). A total of 441 patients with EH (male/female: 236/205, mean age: 50.7 ± 13.8) and 83 control patients underwent 24-hour ambulatory BP monitoring and echocardiography. Obese EH patients had higher circadian BP profile with BP variability, wall thickness, and left ventricular mass than nonobese EH patients and controls (all *p*'s <0.05) without gender differences. EFT was higher in female than in male patients (7.0 ± 2.5 versus 5.9 ± 2.2 mm, *p* < 0.001) and higher in the obese female EH group (7.5 ± 2.6 mm) than in the control (6.4 ± 2.8 mm) or nonobese EH group (6.7 ± 2.8 mm) among women, whereas EFT did not vary among males (5.9 ± 1.9 versus 6.0 ± 2.7 versus 5.9 ± 2.4 mm, *p* = 0.937). Multivariate logistic regression analysis demonstrated that the 24-hour mean BP variability was associated with SBP (*p* = 0.018) and EFT (*p* = 0.016) in female patients, but not in male patients. The relationships among circadian BP variability, obesity, and EFT were affected by gender in different manners. EFT may be a more valuable parameter in the evaluation of BP severity and obesity in women than in men.

## 1. Introduction

Obesity is a worldwide phenomenon, with an incidence increasing in parallel with obesity-associated diseases such as insulin resistance, type 2 diabetes mellitus, and hypertension [1]. The combination of obesity and hypertension places patients at a higher risk of hypertensive end-organ damage and vascular events [2, 3]. Blood pressure (BP) is subject to diurnal variation, and studies using ambulatory BP monitoring (ABPM) have demonstrated the clinical significance of disturbances in the diurnal BP profile and have associated nondipping with a progression in end-organ damage [4, 5]. Moreover, a nondipping pattern of BP has been reported to occur in several conditions that are frequently associated with obesity. Although body mass index (BMI) is associated with the severity of ambulatory hypertension and an increase in daytime BP [1], BMI does not reflect body fat distribution.

Epicardial adipose tissue is related to visceral fat, rather than total adiposity, and shares microcirculation with myocardial tissue and coronary vessels [6, 7]. Because elevated BP is associated with ectopic fat accumulation in the intrathoracic and epicardial areas, a possibility of association of epicardial adipose tissue with hypertension as well as diurnal BP patterns [10, 11] has been found in some recent studies [8, 9]. Epidemiological studies have found that epicardial fat is two to three times more common among females than males and that excessive body weight increases the risk of diabetes mellitus (DM) and cardiovascular diseases approximately one-and-a-half to two times higher in women than it does in men [12]. Furthermore, a higher prevalence of hypertension among overweight adults is found in women than in men [13], which may indicate different susceptibilities based on gender. Given these facts, we aimed to investigate the influence of obesity on the association between epicardial fat thickness (EFT) and circadian BP changes in patients with recently diagnosed essential hypertension (EH), with a gender subgroup analysis.

## 2. Methods

### 2.1. Study Population

This cross-sectional observational single-center cohort study included patients who underwent 24 h ABPM and echocardiography between January 2008 and October 2013. Demographic characteristics recorded at the first visit included age, sex, height, weight, medication, smoking history, and other diseases. Blood was drawn for measurement of total serum cholesterol, triglycerides, high-density lipoprotein (HDL) and low-density lipoprotein (LDL) cholesterol, blood glucose, creatinine, uric acid, and high sensitivity C-reactive protein (hs-CRP). Body mass index (BMI) was calculated as the ratio of dry weight in kilograms to height squared (in square meters). This study was approved by the Institutional Review Board. All patients were required to provide written informed consent to participate.

### 2.2. Inclusion Criteria

A total of 441 consecutive patients, who underwent office BP measurements, 24-hour ABPM, and laboratory measurements for cardiovascular risk factors and echocardiography, were analyzed. Following the recommendations of the European Society of Hypertension [14], a normotensive state was defined as a mean daytime ambulatory systolic and diastolic BP <135/85 mmHg by ABPM, associated with an office BP <140/90 mmHg. True HT was assigned if the average daytime BP was higher than 135/85 mmHg and the average nighttime BP was above 120/75 mmHg. Obesity was defined as a BMI above 25 kg/m^2^, as per the Korean Society for the Study of Obesity [15]. Other inclusion criteria included age between 18 and 65 years and normal renal function.

### 2.3. Exclusion Criteria

The patients with the following diseases were excluded from the study: secondary hypertension, significant liver disease, neurologic disorder, malignant disease, valvular heart disease, heart failure, and history of acute coronary syndrome, myocardial infarction, coronary revascularization procedure, carotid revascularization procedure, ischemic leg ulcer, peripheral revascularization, or amputation. Because the presence of type 2 DM is known to be associated with increased EFT regardless of obesity [6, 7], we also excluded overt type 2 DM from the analysis to avoid its possible confounding impact on the association between EFT and circadian BP variability in our hypertensive patients with or without obesity.

### 2.4. Blood Pressure Measurement

Office BP measurements were performed using an automated device (*EASY X 800* (*R/L*), JAWON Medical, Seoul, Korea). Measurements were taken after patients had rested for 10 min in a sitting position, with the arm comfortably placed at the heart level. Two measurements were taken at 5 min intervals. Each set of two measurements was averaged to give the office systolic and diastolic BP. ABPM was performed on each patient's nondominant arm using an automatically oscillometric device (TONOPORT V, PAR Medizintechnik, Berlin, Germany) on a normal working day. All subjects were instructed to rest or sleep between 10:00 pm and 7:00 am (nighttime) and to continue their usual activities between 7:00 am and 10:00 pm (daytime). The accuracy of the device was checked against the standard auscultatory method to ensure that the difference in BP measurements between methods did not exceed 5 mmHg. The device was set to obtain BP readings at 20 min intervals during the day (7:00 am–10:00 pm) and at 40 min intervals during the night (10:00 pm–7:00 am). Only 24-hour recordings including more than 80% of successful recordings were accepted as valid. Each ABPM dataset was first automatically scanned to remove artifactual readings according to preselected editing criteria. Data were edited by omitting all readings of zero, all heart rate readings <20 or >200, diastolic BP readings >150 and <40 mmHg, systolic BP readings >240 and <70 mmHg, and all readings where the difference between systolic and diastolic BPs was less than 10 mmHg. The following ABPM parameters were evaluated: average ambulatory 24-hour systolic and diastolic BP levels, average ambulatory daytime systolic and diastolic BP levels, average ambulatory nighttime systolic and diastolic BP levels, and mean ambulatory 24-hour, daytime, and nighttime BPs. Additionally, for both SBP and DBP, the magnitude of the nocturnal decline in BP (Δ nocturnal decline) was calculated as follows: daytime average BP minus nighttime average; the percentage change in BP from day to night (% day–night BP) was calculated as (daytime BP − nighttime BP) ×100/daytime BP. With this latter data, a normal dipper pattern was diagnosed when the reduction in the average SBP and DBP during the nighttime was ≥10% of the average daytime values. The nocturnal BP status was also assessed and expressed as either nocturnal normotension or hypertension, with nocturnal hypertension defined as a nighttime BP of ≥120/70 mmHg.

### 2.5. Echocardiographic Measurement

Standard two-dimensional (D) and strain echocardiography were performed on all subjects while lying in the left lateral decubitus position using a 3.5 MHz transducer (Philips iE33, Philips Medical Systems, Bothell, WA, USA), and the echocardiography examiners were blinded to patient information. Measurements of the thickness of interventricular septum and posterior wall, the diameter of the LV cavity, and the LV mass index (LVMI) were performed according to American Society of Echocardiography criteria [16]. Pulsed wave Doppler of the transmitral LV inflow was performed in an apical 4-chamber view with the sample volume placed at the level of the mitral valve tips, and the following measurements of global LV diastolic function were determined: peak early (e) and late (a) diastolic mitral flow velocity, and their ratio, e/a; early (Ea) diastolic mitral annular velocity; deceleration time of the e wave; and LV isovolumetric relaxation time (IVRT).

**Figure 1:**
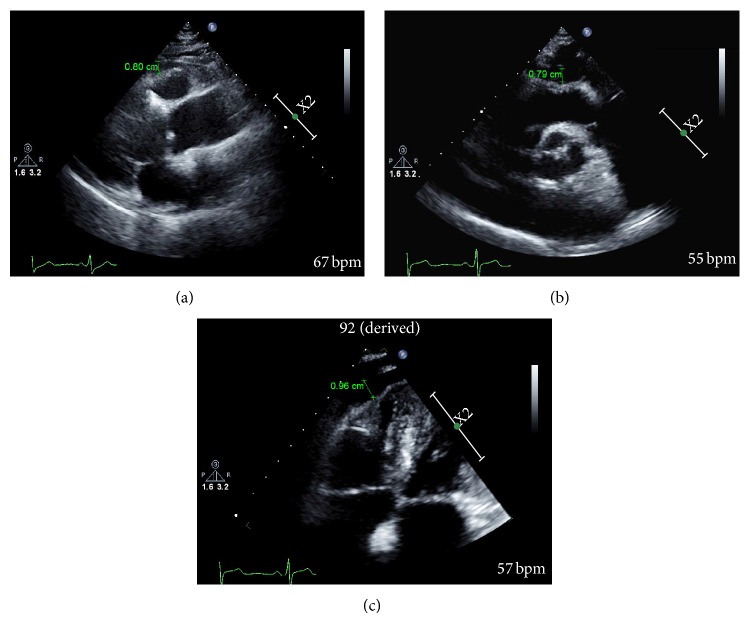
Measurement of epicardial fat thickness (EFT, green arrows) by echocardiography. (a) The maximum EFT was measured perpendicularly from the free wall of the right ventricle at end-systole from the parasternal long axis. (b) Maximum EFT was measured from the point on the free wall of the right ventricle along the midline of the ultrasound beam perpendicular to the aortic annulus as the anatomic landmark. (c) Maximum EFT was measured from the apical 4-chamber focused on the right ventricle echocardiographic images.

Echocardiographic assessments of the EFT were measured perpendicularly from the free wall of the right ventricle at end-systole in three cardiac cycles according to the method previously described by Iacobellis et al. [17] (Figure 1(a)). Epicardial fat was described as the echo-free space between the outer wall of the myocardium and the visceral layer of the pericardium. The maximum EFT was also measured from the point on the free wall of the right ventricle along the midline of the ultrasound beam perpendicular to the aortic annulus as the anatomic landmark (Figure 1(b)) and from the apical 4-chamber view focused on the right ventricle (Figure 1(c)), because one of the critical issues in EFT measurement is the inconsistency in the measurement location, and mean EFT was averaged from the parasternal long axis, parasternal short axis, and apical 4-chamber view images. The offline measurement of EFT was performed by two cardiologists (IK Shim and KI Cho) who were unaware of the clinical data. The measurement of EFT was undergone at least twice for the improvement of reliability. The intra- and interobserver variability of the EFT were 3.2% and 5.6%, respectively.

### 2.6. Statistical Analysis

Statistical analyses were performed with the commercially available computer program SPSS 18.0 for Windows (SPSS Inc., Chicago, IL, USA). Values are expressed as mean ± standard deviation or as a percentage (%). The normality of data was tested using the Kolmogorov-Smirnov test. Parameter differences among the 3 groups were evaluated using the one-way ANOVA test for normally distributed variables and the Kruskal-Wallis test for non-normally distributed variables. Values are expressed as means (standard deviation) if numerical variables or as the number of subjects and their percentages (%), if categorical. Continuous variables were compared using Student's* t*-test. Analysis of categorical data was carried out by Chi-square test. Pearson correlations analysis was performed to determine factors that potentially influenced BP and HR variability. Multivariate logistic regression models for BP variability were built to determine which variables were independently associated with this status. A two-tailed *p* < 0.05 was considered to be statistically significant.

## 3. Results

A total of 441 patients with EH (male/female, 236/205; mean age, 50.7 ± 13.8) and 83 normotensive normal weight patients (controls, male/female, 42/41; mean age, 51.0 ± 13.3) were analyzed, and their clinical features and ambulatory blood pressure parameters according to gender are given in Table 1 (male) and Table 2 (female).

Circadian BP profile and BP variability assessed by 24-hour mean BPs variation were increased in hypertensive patients, especially in hypertensive obese patients, without gender difference (all* p*'s < 0.05). Moreover, the proportion of patients with a nondipping pattern was the highest among the hypertensive obese patients. Although there were no significant differences in HR variability among the groups, hypertensive obese patients had higher daytime and 24-hour mean HR and male hypertensive obese patients also had higher nighttime HR than control patients (all* p*'s < 0.05). Although total cholesterol levels were higher in hypertensive obese patients of both genders than in the controls, male hypertensive obese patients had higher triglycerides and female hypertensive obese patients had lower HDL cholesterol than controls (all* p*'s < 0.05). Although there was no significant difference in systolic function, hypertensive patients showed significantly increased wall thickness and enlarged left atrial diameter, which were more prominent among hypertensive obese patients, without gender difference (Tables 3 and 4). Interestingly, EFT was higher in female than in male patients (7.0 ± 2.5 versus 5.9 ± 2.2 mm, *p* < 0.001). Among women, EFT was highest in the obese EH group (7.5 ± 2.6 mm) whereas that of the control was 6.4 ± 2.8 mm and that of the nonobese EH group was 6.7 ± 2.8 mm; however, EFT did not vary significantly among males (5.9 ± 1.9 versus 6.0 ± 2.7 versus 5.9 ± 2.4 mm, resp., *p* = 0.937).

When we compared the 24-hour ABMP parameters and EFT between male and female patients, female patients had significantly higher EFT (7.0 ± 2.6 versus 5.9 ± 2.2 mm, *p* < 0.001) and a greater daytime BP variation (15.1 ± 4.72 versus 13.6 ± 4.45 mmHg, *p* < 0.001) than male subjects; males had higher 24-hour mean BP (138.8 ± 14.7 versus 137.3 ± 15.3 mmHg, *p* = 0.043) and HR variability (15.6 ± 7.45 versus 14.3 ± 6.02, *p* = 0.023) than female subjects (Table 5).

**Figure 2:**
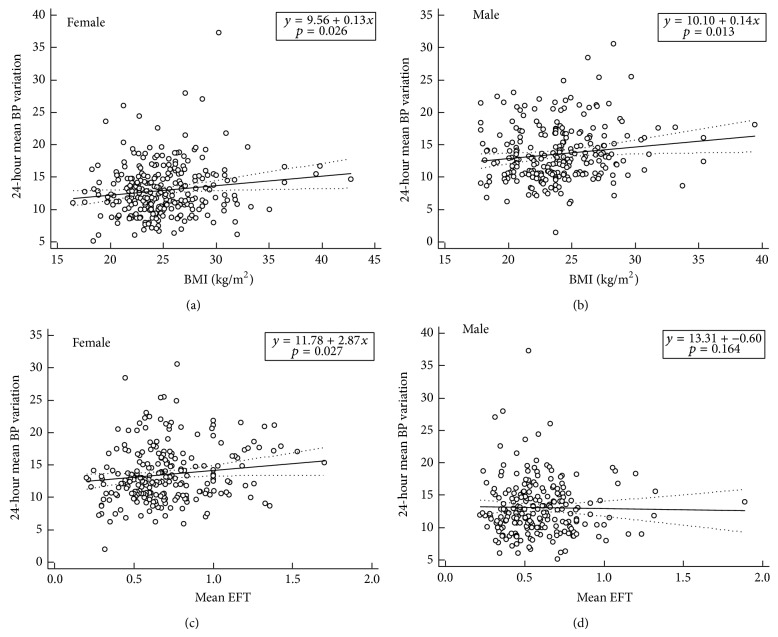
Regression analysis between 24-hour mean blood pressure (BP) difference and body mass index (BMI) or mean epicardial fat thickness (EFT). 24-hour mean BP variation was significantly related to BMI in both females (a) and males (b). However, EFT was significantly correlated with 24-hour mean BP variation only in females (c), not in males (d).

Variable circadian BP profiles and LA size were significantly related to BMI in both females and males (all* p*'s < 0.05, Figures 2(a) and 2(b), Table 6). However, EFT was significantly correlated with 24-hour mean BP (both day and night) as well as variability (mainly night) only in female patients (Figure 2(c)). In addition, EFT was significantly correlated with LA diameter only in female patients. When we investigated the association of quotient of EFT/BMI with BP values, EFT/BMI showed positive correlation with 24-hour systolic BP (*r* = 0.172; *p* = 0.009), 24-hour mean BP (*r* = 0.134; *p* = 0.041), day systolic BP (*r* = 0.150; *p* = 0.022), day systolic BP variation (*r* = 0.133; *p* = 0.043), night systolic BP (*r* = 0.217; *p* = 0.001), night systolic BP variation (*r* = 0.181; *p* = 0.006), night diastolic BP (*r* = 0.132; *p* = 0.047), and night diastolic BP variation (*r* = 0.179; *p* = 0.007). However, no correlation was shown between EFT/BMI and 24-hour ABPM parameters in male. Multivariate logistic regression analysis demonstrated that the 24-hour mean BP variability was associated with SBP (standardized *β* coefficient = 0.199; *p* = 0.018) and EFT (standardized *β* coefficient = 0.175; *p* = 0.016) in female patients, but not in male ones (Table 7). When we performed binary logistic regression analysis to identify the independent determinants of nocturnal nondipping BP pattern, EFT was independent contributor to the nondipping pattern only in female (odds ratio 7.034, 95% confidence interval 2.258 to 21.909, *p* = 0.001) (Table 8).

## 4. Discussion

The most relevant information obtained from this study is that relationships among EFT, obesity, and circadian BP variability are affected by gender in different manners. Although circadian BP profile and BP variability were increased in hypertensive obese patients without gender differences, female patients had significantly higher EFT and greater daytime BP variability than male subjects. BP variability was associated with SBP and EFT only in female patients; therefore, EFT may be a more valuable parameter in the evaluation of BP severity and obesity in women than in men.

The severity of hypertension increased significantly with the degree of obesity. BMI is known to be associated with the severity of ambulatory hypertension and increased daytime BP [3]. In the present study, we investigated the relationship between the parameters derived from 24-hour ABPM (24-hour SBP and DBP, and BP variability) in nondiabetic hypertensive patients with or without obesity. In general, BMI is correlated with BP level, and individuals with abdominal obesity have increased BP levels or are at risk for hypertension [3, 5]. The mechanism behind obesity-related BP elevation has not been fully established. Obesity may induce autonomic dysfunction secondary to the elevation of plasma insulin concentration, because hyperinsulinemia has been associated with sympathetic activation [18, 19] and marked increases in HR [20]. Vice versa, sympathetic hyperactivity is also an independent contributor to the insulin resistance associated with obesity [21, 22]. Thus, it is possible that mean arterial pressure and nighttime HR might remain at daytime levels as a consequence of sustained elevation of plasma insulin concentration. Our hypertensive obese patients had higher proportions of nondipping patterns and higher daytime and 24-hour mean HR; moreover, male hypertensive obese patients also had higher nighttime HR than the controls. These findings are similar to the report of Antic et al. [23], who showed that the normal dipping pattern of BP and HR is rapidly lost following a switch to a high fat diet, without affecting daytime values. Moreover, obesity suppresses nighttime parasympathetic activity and increases nighttime HR values [24]. Because diurnal variations in mean arterial pressure and HR appear to be autonomically mediated, the loss of nocturnal dipping of mean arterial pressure and HR in our obese patients must represent an underlying change in autonomic function. Since the nighttime mean BP was altered in obese patients, the presence of hypertension in obese patients might be missed by office BP measurements that were made only during the daytime. Thus, our results emphasize the importance of continuous 24-hour monitoring of cardiovascular variables, especially in obese patients.

Regarding gender differences, obese females showed a higher tendency towards BP variability than obese males. One interesting finding was that females showed a significant relationship between increased EFT, obesity, and BP variability, but this was not the case in males. This significance still remained after multivariate regression analysis (*p* = 0.016). Still yet, there is no consensus in the literature on the impact of gender on the amount of epicardial fat. Iacobellis et al. showed bigger size of EFT in males than in females [17], and our result showed the greater values of EFT in females. Recent study by Akilli et al. [25] showed larger EFT in females than in males. Thus, these findings may indicate different gender susceptibilities related to obesity and the regional differences in fat distribution, especially EFT distribution. Several studies have reported gender differences regarding the effects of obesity and change in body weight on BP [13, 26] and cardiovascular outcome [12, 27]. It has been reported that obese women have a higher relative risk of diabetes and heart failure with increasing abdominal circumference than men [12] and that obesity has an association with diastolic dysfunction and LVMI only in women [27]. From these results, women can be considered to be more susceptible to cardiovascular adverse effects related to obesity. In our study, accentuated BP variability in obese hypertensive women may have indicated a role in cardiovascular vulnerability. However, the exact explanation for these gender differences is still unclear. One possible mechanism is that such gender differences result from abrupt hormonal and body fat composition changes after menopause [13]. Thus, obese females have a greater amount of visceral adipose tissue which may lead to insulin resistance [28]. Insulin resistance and changes in the autonomic nervous system as a result of redistribution of body fat may have an impact on the cardiovascular system in obese women. Excess fat has traditionally been understood to be found in intra-abdominal organs such as the liver and in subcutaneous tissue. Recently, however, epicardial adipose tissue has been found to reflect visceral adiposity and has been proposed as a new cardiometabolic risk factor [29]. Increased plasma fatty acid levels may stimulate the cardiac autonomic nervous system activity through an increase in plasma catecholamine concentrations, which may be related to impaired diurnal BP patterns [30]. Several reports have found a relationship between BP pattern and EFT [10, 31], and echocardiographically assessed elevated EFT was independently associated with impaired diurnal blood pressure profiles in hypertensive individuals [31]. Also, increased EFT has been reported to be associated with diastolic dysfunction and LA dilatation due to local or systemic effects in untreated hypertensive patients [10]. In our results, variable circadian BP profiles and LA size were significantly related toBMI in both males and females, but EFT was significantly correlated with LA diameter and 24-hour mean BP with variability as well as nocturnal nondipping BP pattern, only in female patients. The present study suggests that there would be a sex-dependent regulation of BP associated with epicardial adipose tissue, and a recent study demonstrated a sex-dependent regulation of diet-induced LVH associated with sexual dimorphic expression of adipocytokines in epicardial adipose tissue [32].

This study has some potential shortcomings. Although our patient sample seems sufficiently large compared to other studies in the literature, in this cross-sectional study we observed predictive factors and outcome variables simultaneously and had no follow-up data. Thus, it was difficult to establish any causal or temporal relationship. Moreover, although we suggested that the hyperinsulinemia would be the possible main cause of elevated nighttime HR levels, we did not measure the plasma insulin levels. Because both pre- and postmenopausal women were included in the study, we cannot assume the impact of estrogen on epicardial fat. Finally, all participants were Korean, and we thus cannot generalize our results to populations of other ethnic groups. In conclusion, the relationship among EFT, obesity, and circadian BP variability was affected by gender in different manners. EFT may be a more valuable parameter in the evaluation of BP severity and obesity in women than in men.

## Figures and Tables

**Table 1:** The clinical characteristics and ambulatory blood pressure measurements of the study population in males.

	Without hypertension	With essential hypertension		ANOVA
	Normal weight (*n* = 41)	Normal weight (*n* = 119)	Obese (*n* = 117)	*p* value
Age (years)	49.4 ± 14.4	49.5 ± 16.7	45.7 ± 12.9	0.177
Body mass index (kg/m^2^)	22.7 ± 1.69	22.5 ± 1.81	28.2 ± 3.16^∗,+^	<0.001
Office systolic BP (mmHg)	126.5 ± 18.4	138.8 ± 19.9	142.3 ± 15.8^∗^	0.027
Office diastolic BP (mmHg)	76.3 ± 16.7	79.3 ± 11.9	82.0 ± 12.8^∗^	0.003
Fasting blood sugar (mg/dL)	98.1 ± 11.1	100.5 ± 17.2	104.5 ± 29.5	0.493
Hemoglobin (mg/dL)	13.9 ± 1.64	14.4 ± 1.55	14.9 ± 1.33^∗^	0.002
Total cholesterol (mg/dL)	171.9 ± 40.3	177.2 ± 37.0	185.5 ± 35.7^∗^	0.016
LDL-cholesterol (mg/dL)	104.2 ± 38.6	93.5 ± 38.9	93.5 ± 38.9	0.064
HDL-cholesterol (mg/dL)	50.1 ± 15.8	50.2 ± 13.8	47.0 ± 12.7	0.071
Triglyceride (mg/dL)	109.9 ± 54.0	119.6 ± 65.1	155.3 ± 92.2^∗,+^	<0.001
HR daytime (/min)	74.2 ± 8.68	76.6 ± 10.4	79.9 ± 13.5^∗^	0.123
BPs daytime (mmHg)	121.3 ± 6.91	144.0 ± 12.9^∗^	146.6 ± 13.3^∗^	<0.001
BPd daytime (mmHg)	76.9 ± 3.87	92.8 ± 9.98^∗^	96.4 ± 11.6^∗,+^	<0.001
HR nighttime (/min)	60.8 ± 8.52	63.9 ± 9.98	66.5 ± 10.1^∗^	0.005
BPs nighttime (mmHg)	111.5 ± 8.21	133.7 ± 14.6^∗^	133.7 ± 14.4^∗^	<0.001
BPd nighttime (mmHg)	69.1 ± 4.69	83.1 ± 9.90^∗^	84.6 ± 13.8^∗^	<0.001
24-hour mean HR (/min)	70.6 ± 8.18	72.3 ± 12.9	76.3 ± 11.3^∗,+^	0.014
24-hour mean HR SD	15.5 ± 5.52	15.1 ± 8.69	16.1 ± 6.65	0.397
24-hour systolic BP (mmHg)	118.6 ± 6.66	141.3 ± 12.4^∗^	143.4 ± 13.1^∗^	<0.001
24-hour diastolic BP (mmHg)	74.7 ± 3.29	90.4 ± 9.36^∗^	93.7 ± 11.2^∗,+^	<0.001
24-hour systolic BP SD	13.7 ± 4.23	14.6 ± 4.07	15.3 ± 4.36^∗^	0.138
24-hour diastolic BP SD	11.0 ± 3.61	12.8 ± 3.84^∗^	13.7 ± 4.67^∗^	0.002
Nondipper	3 (7.3%)	50 (42%)^∗^	62 (53.0%)^∗,+^	<0.001

All values are presented as the mean ± SD. EFT: epicardial fat thickness; BP: blood pressure; LDL: low density lipoprotein; HDL: high density lipoprotein; HR: heart rate; BPs: systolic blood pressure; SD: standard deviation; BPd: diastolic blood pressure; ^∗^ *p* < 0.05 versus normotensive normal weight; ^+^ *p* < 0.05 versus hypertensive normal weight.

**Table 2:** The clinical characteristics and ambulatory blood pressure measurements of the study population in females.

	Without hypertension	With essential hypertension		ANOVA
	Normal weight (*n* = 43)	Normal weight (*n* = 129)	Obese (*n* = 76)	*p* value
Age (years)	52.9 ± 12.2	53.6 ± 11.9	54.7 ± 11.0	0.680
Body mass index (kg/m^2^)	21.9 ± 2.03	22.3 ± 1.92	27.8 ± 2.58^∗,+^	<0.001
Office systolic BP (mmHg)	126.7 ± 17.9	132.9 ± 16.1^∗^	139.8 ± 15.7^∗^	0.036
Office diastolic BP (mmHg)	77.1 ± 11.4	80.7 ± 12.8	82.6 ± 14.3^∗^	0.043
Fasting blood sugar (mg/dL)	94.0 ± 9.48	100.8 ± 24.4	99.3 ± 24.2	0.422
Hemoglobin (mg/dL)	13.2 ± 1.23	13.4 ± 1.59	13.5 ± 1.56	0.608
Total cholesterol (mg/dL)	180.8 ± 47.6	184.8 ± 42.7	196.4 ± 40.8^∗^	0.126
LDL-cholesterol (mg/dL)	105.0 ± 42.3	110.2 ± 32.8	110.0 ± 35.2	0.790
HDL-cholesterol (mg/dL)	53.5 ± 12.9	47.6 ± 11.2^∗^	44.4 ± 9.33^∗,+^	0.001
Triglyceride (mg/dL)	124.0 ± 64.0	135.5 ± 89.0	143.5 ± 89.1	0.571
HR daytime (/min)	72.2 ± 13.9	76.0 ± 13.0	78.2 ± 9.38^∗^	0.014
BPs daytime (mmHg)	119.8 ± 7.89	143.5 ± 12.8^∗^	145.4 ± 14.6^∗^	<0.001
BPd daytime (mmHg)	75.0 ± 5.13	92.1 ± 9.51^∗^	93.3 ± 11.6^∗^	<0.001
HR nighttime (/min)	61.7 ± 6.47	66.1 ± 11.3	65.2 ± 9.95	0.052
BPs nighttime (mmHg)	108.6 ± 20.0	133.2 ± 15.0^∗^	133.7 ± 16.8^∗^	<0.001
BPd nighttime (mmHg)	67.3 ± 6.06	82.2 ± 12.6^∗^	82.3 ± 11.2^∗^	<0.001
24-hour mean HR (/min)	70.6 ± 7.26	73.9 ± 9.49	75.4 ± 9.54^∗^	0.015
24-hour mean HR variation	14.3 ± 6.58	14.2 ± 5.18	14.3 ± 7.03	0.982
24-hour systolic BP (mmHg)	117.7 ± 7.78	140.7 ± 12.4^∗^	142.2 ± 14.7^∗^	<0.001
24-hour diastolic BP (mmHg)	72.6 ± 4.60	89.6 ± 8.93^∗^	90.4 ± 11.0^∗^	<0.001
24-hour systolic BP variation	14.1 ± 4.02	15.8 ± 4.34^∗^	17.1 ± 4.49^∗^	0.002
24-hour diastolic BP variation	11.0 ± 3.66	13.3 ± 4.28^∗^	14.5 ± 4.92^∗^	<0.001
Nondipper	3 (7%)	56 (43.4%)^∗^	39 (51.3%)^∗,+^	<0.001

All values are presented as the mean ± SD. EFT: epicardial fat thickness; BP: blood pressure; LDL: low density lipoprotein; HDL: high density lipoprotein; HR: heart rate; BPs: systolic blood pressure; BPd: diastolic blood pressure; ^∗^ *p* < 0.05 versus normotensive normal weight; ^+^ *p* < 0.05 versus hypertensive normal weight.

**Table 3:** The echocardiographic measurements of the study population in males.

	Without hypertension	With essential hypertension		ANOVA
	Normal weight (*n* = 41)	Normal weight (*n* = 119)	Obese (*n* = 117)	*p* value
EFT (mm)	6.0 ± 2.7	5.9 ± 2.4	5.9 ± 1.9	0.937
LVEDD (cm)	46.6 ± 4.92	45.9 ± 4.97	46.8 ± 5.16	0.655
LVESD (cm)	29.6 ± 4.45	29.0 ± 4.66	30.0 ± 4.87	0.475
IVSTd (mm)	11.7 ± 2.73	12.8 ± 2.92	13.5 ± 2.59^∗^	0.007
PWTd (mm)	9.72 ± 1.65	10.7 ± 1.80^∗^	11.6 ± 2.26^∗,+^	<0.001
RWT	0.42 ± 0.10	0.47 ± 0.11^∗^	0.50 ± 0.13^∗^	0.016
LVMI (g/m^2^)	103.0 ± 39.2	115.2 ± 30.8	118.3 ± 32.1	0.106
LA diameter	33.3 ± 4.62	33.8 ± 4.69	36.3 ± 5.79^∗^	0.002
LA volume	15.3 ± 4.84	15.3 ± 4.76	17.1 ± 5.55^∗,+^	0.043
Aorta diameter	32.8 ± 4.42	33.3 ± 4.06	34.2 ± 3.70	0.080
EF (%)	66.2 ± 7.17	66.3 ± 8.98	65.3 ± 8.23	0.437
E (cm/sec)	66.2 ± 16.9	65.5 ± 14.8	65.0 ± 15.8	0.339
E/Ea	8.7 ± 2.72	9.2 ± 3.21	9.8 ± 3.65	0.123
A (cm/sec)	67.4 ± 20.9	67.8 ± 21.0	68.2 ± 18.7	0.991

All values are presented as the mean ± SD. EFT: epicardial fat thickness; LVDEDD: left ventricular end-diastolic diameter; LVESD: left ventricular end-systolic diameter; IVSTd: diastolic interventricular septal wall thickness; PWTd: diastolic posterior wall thickness; LVMI: left ventricular mass index; LA: left atrial diameter; EF: ejection fraction; E: peak early diastolic mitral filling velocity; Ea: mitral annular velocity; A: peak late diastolic mitral filling velocity; ^∗^ *p* < 0.05 versus normotensive normal weight; ^+^ *p* < 0.05 versus hypertensive normal weight.

**Table 4:** The echocardiographic measurements of the study population in females.

	Without hypertension	With essential hypertension		ANOVA
	Normal weight (*n* = 43)	Normal weight (*n* = 129)	Obese (*n* = 76)	*p* value
EFT (mm)	6.4 ± 2.8	6.7 ± 2.8^∗^	7.5 ± 2.6^∗,+^	0.040
LVEDD (cm)	44.2 ± 6.21	44.9 ± 5.41	44.2 ± 4.40	0.609
LVESD (cm)	27.2 ± 4.42	28.1 ± 4.73	27.0 ± 3.89	0.302
IVSTd (mm)	11.6 ± 2.36	11.8 ± 2.04	12.8 ± 2.23^∗^	0.009
PWTd (mm)	9.79 ± 1.67	10.4 ± 1.97^∗^	10.9 ± 1.66^∗^	0.023
RWT	0.45 ± 0.11	0.47 ± 0.11	0.50 ± 0.09^∗^	0.134
LVMI (g/m^2^)	106.3 ± 29.8	113.6 ± 32.3	111.5 ± 30.2	0.498
LA diameter	33.2 ± 5.89	34.4 ± 5.31	35.3 ± 4.58^∗^	0.051
LA volume	16.5 ± 7.97	17.2 ± 5.97^∗^	17.9 ± 6.63^∗^	0.049
Aorta diameter	31.0 ± 3.53	31.5 ± 3.60	31.7 ± 2.89	0.603
EF (%)	68.7 ± 5.87	67.8 ± 7.00	69.4 ± 5.77	0.284
E (cm/sec)	69.5 ± 19.2	68.7 ± 18.5	66.7 ± 17.1	0.717
E/Ea	9.15 ± 2.76	9.95 ± 2.77	10.6 ± 3.13	0.048
A (cm/sec)	66.9 ± 16.6	70.6 ± 19.6	72.4 ± 15.7	0.367

All values are presented as the mean ± SD. EFT: epicardial fat thickness; LVDEDD: left ventricular end-diastolic diameter; LVESD: left ventricular end-systolic diameter; IVSTd: diastolic interventricular septal wall thickness; PWTd: diastolic posterior wall thickness; LVMI: left ventricular mass index; LA: left atrial diameter; EF: ejection fraction; E: peak early diastolic mitral filling velocity; Ea: mitral annular velocity; A: peak late diastolic mitral filling velocity; ^∗^ *p* < 0.05 versus normotensive normal weight; ^+^ *p* < 0.05 versus hypertensive normal weight.

**Table 5:** Comparison of ambulatory blood pressure parameters between male and female patients.

	Male (*n* = 277)	Female (*n* = 247)	*p* value
EFT (mm)	5.9 ± 2.2	7.0 ± 2.6	<0.001
HR daytime (/min)	77.2 ± 11.6	76.5 ± 11.6	0.761
BPs daytime (mmHg)	141.7 ± 15.1	140.1 ± 15.7	0.218
BPs daytime variation (mmHg)	13.6 ± 4.45	15.1 ± 4.72	<0.001
BPd daytime (mmHg)	92.0 ± 12.0	89.5 ± 11.7	0.019
BPd daytime variation (mmHg)	12.1 ± 4.73	12.7 ± 5.02	0.196
HR nighttime (/min)	64.5 ± 10.0	65.0 ± 10.3	0.555
BPs nighttime (mmHg)	130.4 ± 15.8	129.1 ± 18.9	0.388
BPs nighttime variation (mmHg)	12.0 ± 4.23	12.4 ± 3.94	0.215
BPd nighttime (mmHg)	81.7 ± 12.4	79.7 ± 12.6	0.074
BPd nighttime variation (mmHg)	10.2 ± 4.11	9.97 ± 3.81	0.608
24-hour mean HR (/min)	73.7 ± 11.8	74.1 ± 9.30	0.680
24-hour mean HR variation	15.6 ± 7.45	14.3 ± 6.02	0.023
24-hour mean BP (mmHg)	138.8 ± 14.7	137.3 ± 15.3	0.043
24-hour mean BP variation (mmHg)	105.6 ± 12.1	103.4 ± 12.0	0.079
24-hour systolic BP (mmHg)	13.0 ± 4.02	13.6 ± 4.27	0.204
24-hour diastolic BP (mmHg)	89.4 ± 11.5	86.9 ± 11.2	0.012
24-hour systolic BP variation	14.8 ± 4.24	15.9 ± 4.43	0.002
24-hour diastolic BP variation	12.9 ± 4.27	13.3 ± 4.52	0.289

All values are presented as the mean ± SD. EFT: epicardial fat thickness; HR: heart rate; BPs: systolic blood pressure; BPd: diastolic blood pressure; BP: blood pressure.

**Table 6:** Correlation between body mass index or epicardial fat thickness and 24-hour ambulatory blood pressure parameters.

	Body mass index		Epicardial fat thickness	
	*r*	*p*	*r*	*p*
Male				
24-hour mean BP	0.166	0.006	−0.052	0.437
24-hour mean BP variation	0.137	0.022	−0.019	0.773
Day mean BP	0.163	0.007	−0.039	0.560
Day mean BP variation	0.147	0.014	−0.046	0.495
Night mean BP	0.024	0.696	−0.068	0.324
Night mean BP variation	0.128	0.037	0.047	0.491
24-hour mean HR	0.109	0.071	0.109	0.106
24-hour mean HR variation	0.106	0.080	0.007	0.919
Day HR	0.061	0.314	0.084	0.213
Day HR variation	0.172	0.004	0.008	0.907
Night HR	0.103	0.092	0.033	0.627
Night HR variation	0.043	0.482	−0.071	0.302
Left atrial diameter	0.359	<0.001	−0.021	0.766
Relative wall thickness	0.116	0.091	0.037	0.605

Female				
24-hour mean BP	0.200	0.002	0.195	0.005
24-hour mean BP variation	0.142	0.026	0.121	0.040
Day mean BP	0.214	0.001	0.191	0.006
Day mean BP variation	0.120	0.059	0.121	0.080
Night mean BP	0.124	0.054	0.182	0.009
Night mean BP variation	0.002	0.979	0.165	0.018
24-hour mean HR	0.080	0.210	0.000	0.998
24-hour mean HR variation	0.082	0.198	−0.075	0.281
Day HR	0.030	0.642	−0.032	0.641
Day HR variation	0.084	0.187	−0.075	0.281
Night HR	0.092	0.154	0.015	0.823
Night HR variation	−0.080	0.216	0.081	0.245
Left atrial diameter	0.186	0.004	0.159	0.012
Relative wall thickness	0.144	0.040	0.094	0.175

BP: blood pressure; HR: heart rate.

**Table 7:** Multiple linear regression analysis for the 24-hour mean BP variation in study groups according to the gender.

	Univariate analysis				Multivariate analysis	
	Coefficient (*β*)	95% CI	*p*	Coefficient (*β*)	95% CI	*p*
Age	−0.082	−0.057 to 0.010	0.173	−0.057	−0.062 to 0.026	0.428
Systolic blood pressure	0.258	0.041 to 0.108	<0.001	0.134	−0.035 to 0.118	0.288
Diastolic blood pressure	0.256	0.052 to 0.137	<0.001	0.096	−0.061 to 0.136	0.457
LDL-cholesterol	−0.055	−0.024 to 0.010	0.417	−0.036	−0.022 to 0.013	0.601
HDL-cholesterol	−0.003	−0.044 to 0.042	0.968	−0.014	−0.048 to 0.040	0.846
Triglycerides	−0.066	−0.011 to 0.004	0.323	−0.095	−0.013 to 0.002	0.179
Hemoglobin	0.241	−0.332 to 0.425	0.809	−0.022	−0.518 to 0.383	<0.001
Body mass index	0.149	0.036 to 0.303	0.013	0.115	−0.048 to 0.283	0.096
Epicardial fat thickness	0.100	−0.003 to 0.007	0.164	−0.043	−3.528 to 1.808	0.526

24-hour mean BP variation in males (*R* ^2^ = 0.105, adjusted *R* ^2^ = 0.065 in multivariate analysis)						

Age	0.104	−0.008 to 0.084	0.104	0.110	−0.018 to 0.100	0.175
Systolic blood pressure	0.289	0.047 to 0.114	<0.001	0.199	0.002 to 0.020	0.018
Diastolic blood pressure	0.315	0.075 to 0.166	<0.001	0.212	−0.027 to 0.211	0.127
LDL-cholesterol	0.094	−0.661 to 3.602	0.175	0.155	−0.15 to 5.784	0.063
HDL-cholesterol	0.104	−0.016 to 0.087	0.173	−0.038	−0.098 to 0.074	0.778
Triglycerides	0.122	−0.001 to 0.014	0.107	0.086	−0.030 to 0.089	0.328
Hemoglobin	0.071	−0.219 to 0.621	0.346	0.047	−0.331 to 0.602	0.566
Body mass index	0.142	0.022 to 0.337	0.026	0.140	−0.029 to 0.405	0.089
Epicardial fat thickness	0.170	0.002 to 0.039	0.027	0.175	0.002 to 0.042	0.033

24-hour mean BP variation in females (*R* ^2^ = 0.177, adjusted *R* ^2^ = 0.122 in multivariate analysis)						

LDL: low density lipoprotein; HDL: high density lipoprotein; HR: heart rate; BP: blood pressure; CI: confidence interval.

**Table 8:** Binary logistic regression analysis to identify the independent determinants of nocturnal nondipping blood pressure (BP) pattern.

	Odds ratio	95% confidence interval	*p*
Age	1.003	0.981 to 1.025	0.782
Epicardial fat thickness	0.499	0.126 to 1.974	0.322
Body mass index	1.061	0.974 to 1.157	0.177
Hypertension	12.708	2.911 to 55.479	0.001
Dyslipidemia	1.019	0.562 to 1.848	0.950

Presence of nondipping BP pattern in males (adjusted *R* ^2^ = 0.163)			

Age	1.003	0.978 to 1.030	0.792
Epicardial fat thickness	7.034	2.258 to 21.909	0.001
Body mass index	0.940	0.857 to 1.031	0.940
Hypertension	6.479	2.462 to 17.053	<0.001
Dyslipidemia	1.173	0.648 to 2.122	0.598

Presence of nondipping BP pattern in females (adjusted *R* ^2^ = 0.176)			
